# Chemotherapy-induced neutropenia and treatment efficacy in advanced non-small-cell lung cancer: a pooled analysis of 6 randomized trials

**DOI:** 10.1186/s12885-021-08323-4

**Published:** 2021-05-14

**Authors:** Piera Gargiulo, Laura Arenare, Cesare Gridelli, Alessandro Morabito, Fortunato Ciardiello, Vittorio Gebbia, Paolo Maione, Alessia Spagnuolo, Giuliano Palumbo, Giovanna Esposito, Carminia Maria Della Corte, Floriana Morgillo, Gianfranco Mancuso, Raimondo Di Liello, Adriano Gravina, Clorinda Schettino, Massimo Di Maio, Ciro Gallo, Francesco Perrone, Maria Carmela Piccirillo

**Affiliations:** 1 Clinical Trials Unit, Istituto Nazionale Tumori, IRCCS, Fondazione G. Pascale, Via Mariano Semmola, 80131 Naples, Italy; 2 Division of Medical Oncology, Ospedale “S.G. Moscati”, Contrada Amoretta, 83100 Avellino, Italy; 3 Thoracic Medical Oncology, Istituto Nazionale Tumori, IRCCS, Fondazione G. Pascale, Via Mariano Semmola, 80131 Naples, Italy; 4 Department of Precision Medicine, Medical Oncology, Università degli Studi della Campania “Luigi Vanvitelli”, Via S. Pansini 5, 80131 Naples, Italy; 5 La Maddalena Clinic for Cancer, Department Promise, Medical Oncology, Università di Palermo, 90100 Palermo, Italy; 6 Department of Oncology, University of Turin, Ordine Mauriziano Hospital, Via Magellano 1, 10028 Turin, Italy; 7 Medical Statistics, Università degli Studi della Campania “Luigi Vanvitelli”, Via L. Armanni, 80128 Napoli, Italy

**Keywords:** Lung cancer, Chemotherapy-induced neutropenia (CIN), Retrospective-prospective design, Prognostic factors, Overall survival

## Abstract

**Background:**

Chemotherapy-induced neutropenia (CIN) has been demonstrated to be a prognostic factor in several cancer conditions. We previously found a significant prognostic value of CIN on overall survival (OS), in a pooled dataset of patients with advanced non-small-cell lung cancer (NSCLC) receiving first line chemotherapy from 1996 to 2001. However, the prognostic role of CIN in NSCLC is still debated.

**Methods:**

We performed a post hoc analysis pooling data prospectively collected in six randomized phase 3 trials in NSCLC conducted from 2002 to 2016. Patients who never started chemotherapy and those for whom toxicity data were missing were excluded. Neutropenia was categorized on the basis of worst grade during chemotherapy: absent (grade 0), mild (grade 1–2), or severe (grade 3–4). The primary endpoint was OS. Multivariable Cox model was applied for statistical analyses. In the primary analysis, a minimum time (landmark) at 180 days from randomization was applied in order to minimize the time-dependent bias.

**Results:**

Overall, 1529 patients, who received chemotherapy, were eligible; 572 of them (who received 6 cycles of treatment) represented the landmark population. Severe CIN was reported in 143 (25.0%) patients and mild CIN in 135 (23.6%). At multivariable OS analysis, CIN was significantly predictive of prognosis although its prognostic value was entirely driven by severe CIN (hazard ratio [HR] of death 0.71; 95%CI: 0.53–0.95) while it was not evident with mild CIN (HR 1.21; 95%CI: 0.92–1.58). Consistent results were observed in the out-of-landmark group (including 957 patients), where both severe and mild CIN were significantly associated with a reduced risk of death.

**Conclusion:**

The pooled analysis of six large trials of NSCLC treatment shows that CIN occurrence is significantly associated with a longer overall survival, particularly in patients developing severe CIN, confirming our previous findings.

**Supplementary Information:**

The online version contains supplementary material available at 10.1186/s12885-021-08323-4.

## Introduction

Platinum-based combination chemotherapy represents the historical first-line treatment in patients with advanced non-small-cell lung cancer (NSCLC), without targetable mutations, and good performance status (PS) [1]. In the ‘90s, this strategy became the standard of care due to a small but significant survival advantage in adult patients [1]. Even considering the recent advances in treatment with targeted agents and immune checkpoint inhibitors, most patients are still candidates to receive platinum-based chemotherapy.

Chemotherapy-induced neutropenia (CIN) is one of the most common adverse events reported in cancer patients [2, 3]. Often it is a dose-limiting toxicity leading to treatment delays and/or dose reductions and in case of chemotherapy-induced febrile neutropenia (CIFN) there are increased clinical risks and financial costs related to its diagnosis and treatment [2].

However, CIN appears to be more than an adverse event, and can play a role as a marker for improved outcomes [2–4]. Some studies have showed that CIN is associated with longer survival in various cancer conditions; thus, it was hypothesized that CIN could represent a marker of effective dosing of anticancer drugs. Consequently, the absence of neutropenia may indicate a lack of efficacy of the chemotherapy related to pharmacogenetic variables and differences in drug metabolism. Additionally, it has been suggested that the body surface area (BSA) conventionally used for dosing drugs does not take into account the inter-patient variability in metabolism resulting in over- or under treatment that could be associated with undesirable toxicity and an unpredictable variation in treatment efficacy [5].

In 2005, we performed a pooled analysis of three randomized trials of NSCLC patients who received chemotherapy as first line treatment, in which CIN occurrence, independently of severity, was associated with longer survival [4]. Subsequently, other authors reported data supporting our findings, in patients with completely resected NSCLC [6], and in the metastatic setting for patients receiving gemcitabine/platinum-based chemotherapy [7]. Similar finding was seen in patients treated with cisplatin/docetaxel-based chemotherapy [8]. In contrast, a retrospective analysis did not find a significant relationship between survival and CIN in 190 patients receiving doublet platinum containing chemotherapy in NSCLC [9].

Therefore, we pooled data prospectively collected in further six randomized phase 3 trials to assess whether the CIN occurrence was a significant prognostic factor for advanced NSCLC and possibly confirm our previous findings.

## Material and methods

### Patient population and treatments

A retrospective-prospective study design [10] was applied to multicenter randomized trials promoted by the National Cancer Institute of Naples, between 2002 and 2016, in advanced NSCLC: CALC1 (ClinicalTrials.gov NCT00330746) [11], GECO (ClinicalTrials.gov NCT00385606) [12], TORCH (ClinicalTrials.gov NCT00349219) [13], MILES2 (ClinicalTrials.gov NCT00401492) [14, 15], MILES3 and MILES4(ClinicalTrials.gov NCT01656551) [16]. Primary results have already been published. All these 6 trials were approved by Ethical Committees and all patients gave written informed consent.

MILES2, MILES3 and MILES4 were dedicated to elderly patients (> 70 years old) [14–16]. CALC1 included two different cohorts, one with patients older than 70 and one with adult PS2 patients [11]. GECO was limited to adult patients [12]. All trials tested first-line chemotherapy treatments; the TORCH experimental arm (patients receiving only erlotinib as first-line treatment) was excluded [13]. Patients assigned to study arms testing the combination of chemotherapy with other non-cytotoxic drugs (rofecoxib in the GECO study and cetuximab in CALC-1) were eligible. Planned treatment duration was 6 cycles for all the trials. Details of the characteristics of the six trials are reported in Table S1 online.

For the present analysis, patients were eligible if they had received at least one cycle of chemotherapy and at least one toxicity case report form was available. All cytotoxic treatments were given on days 1 and 8 every 21 days. In MILES4, single-agent pemetrexed and the combination of cisplatin plus pemetrexed (Arm C and D, respectively) were administered on day 1 every 21 days. Minimum eligibility criteria to receive chemotherapy on day 1 and day 8 were also similar across included trials: neutrophil count ≥1.5 × 10^9^/L (≥2.0 × 10^9^/L in MILES2 and MILES3), platelets ≥100 × 10^9^/L, hemoglobin ≥8 g/dL and no grade ≥ 2 non-hematological toxicity (excluding alopecia). If these conditions were not met on day 1 or 8, chemotherapy was postponed or omitted, as required per protocol. Treatment was discontinued in case of unacceptable toxicities, treatment refusal or consent withdrawal.

Management of neutropenia was similar among the trials. All protocols, indeed, suggested the use of granulocyte-colony stimulating factor (G-CSF) in case of grade 4 neutropenia, but did not recommend its prophylactic use. In addition, dose reductions of all drugs were recommended by protocol as a result of toxicities in TORCH, MILES3 and MILES4, whereas no dose reductions were planned in GECO and MILES2. Methods for toxicity data collection and follow-up rules were comparable and were managed at the same trial center, the Clinical Trial Unit of the National Cancer Institute of Naples.

### Neutropenia assessment

Neutropenia was codified according to the National Cancer Institute Common Toxicity Criteria (NCI-CTC) version 2.0 in MILES2 and GECO, the Common Terminology Criteria for Adverse Events (CTCAE) version 3.0 in TORCH and the Common Terminology Criteria for Adverse Events (CTCAE) version 4.0 in MILES3 and MILES4*.* However, definition of neutropenia grades does not change between the three scales: grade 1 if absolute neutrophil count [ANC] < 2000/mmc, grade 2 if ANC < 1500/mmc, grade 3 if ANC < 1000/mmc, and grade 4 if ANC < 500/mmc. For the present study, CIN was defined as the worst grade of neutropenia suffered by each patient during study treatment. CIN was then classified into absent (grade 0), mild (grade 1 or 2) or severe (grade 3 or 4).

### Statistical methods

The risk of developing at least 1 episode of neutropenia typically increases over time during treatment, and a time-dependent bias may arise, since patients who develop neutropenia must have survived until the time they developed neutropenia. As in our previous analysis [4], to neutralize this bias, we applied the landmark strategy, where patients censored or having an event before a predefined minimum time (landmark) were excluded from the primary analysis. A landmark time of 180 days was predefined in order to include the maximum expected length of treatment, even accounting for possible delays. Therefore, the primary analysis in the “landmark group” involved only patients who received all six planned cycles of chemotherapy, and who were alive 180 days after randomization.

Patients not eligible in the ‘landmark’ group represented the ‘out-of-landmark’ group.

Baseline characteristics were reported for both populations; the association between categorical variables and CIN grades were tested by Pearson’s Chi Square, while ANOVA was applied for continuous variables.

OS was the primary endpoint, defined as the time from day 181 after randomization to the date of death. Patients not reaching an event were censored at the date of last information on their vital status. As the occurrence of neutropenia is intrinsically affected by the type of treatment administered, all statistical analyses were stratified by treatment arm.

OS curves were estimated using the Kaplan–Meier method and compared with a stratified log rank test. Hazard ratios (HR) of death and 95% confidence intervals (CIs) were estimated with Cox proportional hazards model stratified by treatment group, using age (continuous, increasing), sex, stage (IV vs IIIB), performance status (2 vs 0–1) and histological subtype (adenocarcinoma vs squamous, and other vs squamous) as covariates. HRs were estimated for the two grades of CIN (mild vs absent and severe vs absent), and the overall *p*-value for CIN was calculated by the likelihood ratio test comparing two models, one with and one without CIN covariates.

A secondary analysis was also performed in the out-of-landmark group (patients who received < 6 cycles of chemotherapy, or who received six cycles but died within 180 days of randomization). OS was defined as the time from randomization to the date of death. Patients not reaching an event were censored at the date of last information on their vital status. This analysis was stratified not only by treatment arm but also by the number of cycles of treatment received.

All statistical tests were two tailed and *p* values of less than 0.05 were considered as significant.

Statistical analyses were performed using Stata/MP for Windows (version 14.2).

## Results

**Fig. 1:**
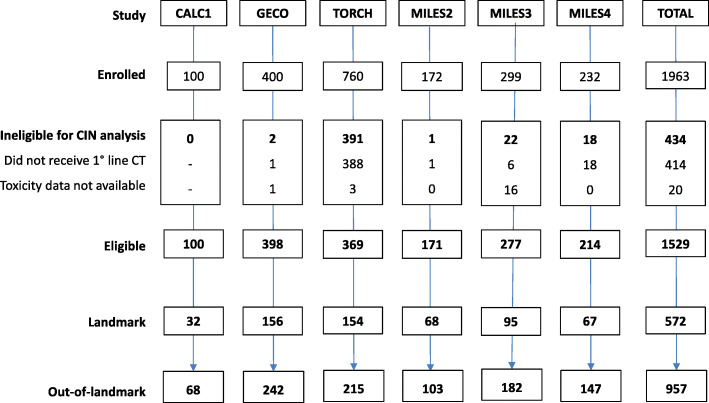
Flowchart of the individual patient data analysis

Out of the 1963 patients initially enrolled in the trials, a total of 1529 subjects, who received chemotherapy and had toxicity information, were pooled from the CALC1, GECO, TORCH, MILES2, MILES3 and MILES4 trials and analyzed. Among them, 572 patients were included in the landmark population and 957 patients (who received < 6 cycles or died before 180 days) remained in the out-of-landmark population (Fig. 1). The distribution of patients according to trial and treatment in the whole population and in landmark and out-of-landmark groups is reported in Table S2 online.

In the whole eligible population, the median age was 69.7 years (range 61.0–74.5), and three quarters of patients were male. Most patients had a PS of 0–1, a metastatic disease, and percentages of squamous and non-squamous histology were balanced (Table 1). But for PS (as expected), baseline characteristics were similar among patients in the landmark and out-of-landmark populations. The planned number of 6 cycles of treatment was reached in 42.4% of patients in the whole population.

**Table 1:** Patient characteristics in the eligible/landmark/out-of-landmark populations

	Whole eligible population (***N*** = 1529)	Landmark population (***N*** = 572)	Out-of-landmark population (***N*** = 957)
**Age**. median (IQR)			
	69.7 (61.0–74.5)	68.6 (60.5–73.9)	70.1 (61.2–74.7)
**Gender.** n (%)			
Male	1172 (76.7%)	427 (74.7)	745 (77.8%)
Female	357 (23.3%)	145 (25.3)	212 (22.2%)
**Performance status**. n (%)			
0–1	1480 (96.8%)	557 (97.4)	923 (96.4%)
2	49 (3.2%)	15 (2.6)	34 (3.6%)
**Stage**. n (%)			
IIIb	179 (11.7%)	72 (12.6)	107 (11.2%)
IV	1350 (88.3%)	500 (87.4)	850 (88.8%)
**Histology**. n (%)			
Squamous	619 (40.5%)	213 (37.2)	406 (42.4%)
Non squamous	674 (44.1%)	275 (48.1)	399 (41.7%)
Undefined	236 (15.4%)	84 (14.7)	152 (15.9%)
**Chemotherapy cycles**. n (%)			
1	209 (13.7%)	–	209 (21.8%)
2	147 (9.6%)	–	147 (15.4%)
3	327 (21.4%)	–	327 (34.2%)
4	108 (7.1%)	–	108 (11.3%)
5	89 (5.8%)	–	89 (9.3%)
6	649 (42.4%)	572 (100.0)	77 (8.0%)

The worst recorded grade of neutropenia in the various groups is reported in Table 2. In the landmark group, mild neutropenia (grade 1–2) occurred in 135 (23.6%) of 572 patients and severe neutropenia (grade 3–4) in 143 (25.0%), while rates were lower (13.9 and 13%, respectively) in the out-of-landmark group. Characteristics of patients according to grade of neutropenia are reported in Tables S3, S4 and S5 online. Patients with neutropenia were younger, less frequently with poor PS and squamous histology compared with those without neutropenia, without remarkable differences between the landmark and out-of-landmark populations.

**Table 2:** Worst grade of neutropenia in the analyzed populations

	Landmark population (***N*** = 572)	Out-of-landmark population (***N*** = 957)
**Worst grade of Neutropenia**. n (%)		
0	294 (51.4)	705 (73.7)
1	47 (8.2)	56 (5.9)
2	88 (15.4)	72 (7.5)
3	100 (17.5)	86 (9.0)
4	43 (7.5)	38 (4.0)

The higher incidence of severe neutropenia was observed with cisplatin plus gemcitabine regimen, in particular it was 35.9% in the TORCH study (Table S6).

Figure S1 shows a relatively equal distribution of worst grade of neutropenia over cycles in the landmark population.

**Fig. 2:**
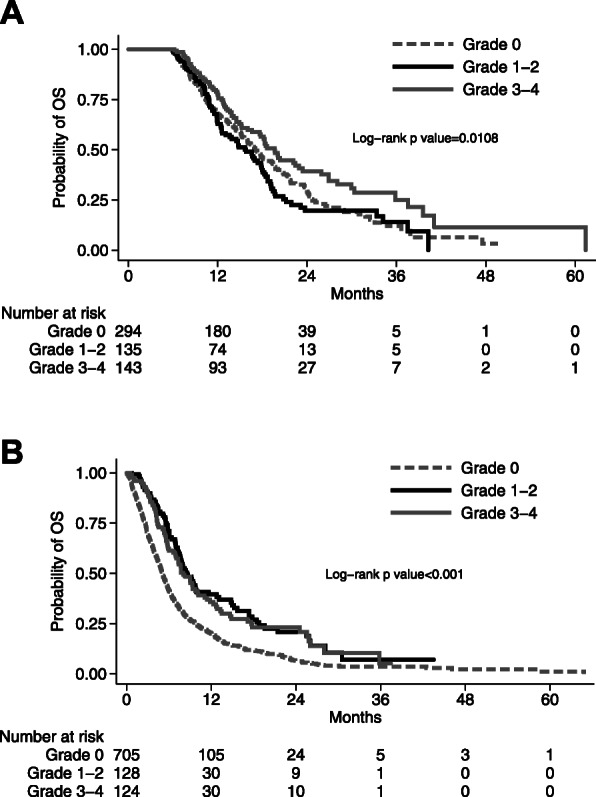
Overall survival by grade of neutropenia for patients in the landmark population (**a**) and in the out-of-landmark population (**b**)

In the landmark group, with a median follow-up of 23.4 months, 358/572 patients (62.6%) died (Fig. 2a). Median OS was 17.0 months (95% CI: 15.3–19.3) for patients without neutropenia, 15.7 (95% CI: 12.5–17.9) for those with mild neutropenia, and 19.6 months (95% CI: 16.6–23.4) for those with severe neutropenia. The association of severe neutropenia with a lower mortality was confirmed in the multivariable analysis (HR 0.71, 95%CI: 0.53–0.95) while it was not evident with mild neutropenia (HR 1.21, 95%CI: 0.92–1.58; Table 3).

**Table 3:** Multivariable analysis of death in the landmark population

	Landmark population (358 events / 572 patients)		
	HR^a^	(95% CI)	***P***
**Chemotherapy-induced neutropenia**			0.048
Grade 1–2 vs 0	1.21	(0.92–1.58)	
Grade 3–4 vs 0	0.71	(0.53–0.95)	
**Age** (continuous)	1.03	(1.01–1.05)	0.002
**Gender**			0.066
Female vs Male	0.77	(0.58–1.01)	
**Performance status**			0.181
2 vs 0–1	1.98	(0.73–5.39)	
**Stage**			0.156
IV vs IIIB	1.29	(0.91–1.82)	
**Histology**			0.13
Non squamous vs Squamous	0.91	(0.68–1.20)	
Undefined vs Squamous	1.27	(0.89–1.81)	

^a^Stratified by treatment arm

In the out-of-landmark group, with at a median follow-up of 21.9 months, 731/957 patients (76.4%) died (Fig. 2b). Median OS was 5.2 (95% CI: 4.8–5.6) for patients without neutropenia, 8.4 (95% CI: 7.4–9.9) in those with mild neutropenia and 7.7 (95% CI: 6.6–9.7) in those with severe neutropenia. This finding was also confirmed in the multivariable analysis, where the mortality benefit appeared for both mild and severe CIN (Table 4).

**Table 4:** Multivariable analysis of death in the out-of-landmark population

	Out-of-landmark population (731 events / 957 patients)		
	HR^a^	(95% CI)	***P***
**Worst grade of neutropenia**			< 0.001
Grade 1–2 vs 0	0.51	(0.40–0.66)	
Grade 3–4 vs 0	0.64	(0.50–0.81)	
**Age** (continuous)	1.00	(0.99–1.01)	0.837
**Gender**			
Female vs Male	0.70	(0.57–0.85)	< 0.001
**Performance status**			
2 vs 0–1	1.48	(0.85–2.58)	0.164
**Stage**			
IV vs IIIB	1.38	(1.06–1.79)	0.017
**Histology**			0.925
Non squamous vs Squamous	0.90	(0.73–1.09)	
Undefined vs Squamous	1.01	(0.80–1.28)	

^a^Stratified by treatment arm and number of cycles

## Discussion

We performed a pooled analysis of six randomized clinical trials in advanced NSCLC and verified the prognostic role of CIN on OS both in the landmark and out-of-landmark populations. Overall, the findings confirm a prognostic role of CIN; particularly, in the landmark population, severe CIN was significantly associated with a lower hazard of death, and in the out-of-landmark population (with more patients and a higher death rate that lead to higher statistical power) both severe and mild CIN were significantly associated with a lower hazard of death.

Large evidence suggests that hematologic toxicities could be a marker of the activities of cytotoxic drugs [17]. Therapeutic benefit depends on the amount of drug achieving the target as well as on the sensitivity of the target itself [4]. A retrospective analysis of elderly patients with advanced NSCLC reported that an adequate dose intensity of chemotherapy has a significant positive impact on both response rate and OS [18].

The dose of cytotoxic drugs is based mainly on BSA (for most drugs) or creatinine clearance (essentially for carboplatin) [19]. However, a poor relation between BSA and pharmacokinetics of anticancer drugs has been reported [5]. The drug availability is affected by significant inter-patient and intra-individual differences in drug clearance irrespective of surface area, linked to pharmacogenetic background (e.g. variability in the activity of drug metabolizing enzymes and transporters), pharmacokinetic interactions due to concomitant medications, and impaired organ function [5]. Hence, a small variation in the administered dose can determine severe and life-threatening toxicity in some patients, and poor antitumor effects in others [19]. In this context, the absence of myelosuppression may be mirror of an inadequate antitumor effect.

In 2005 we performed a pooled analysis of patients with NSCLC treated with chemotherapy within three prospective randomized trials that shared similar protocols and applied a landmark approach (analysis restricted to patients completing six cycles or alive 180 days after randomization) to minimize the time-dependent bias related to the fact that neutropenia could be higher in those receiving more chemotherapy cycles and, thus, higher in those surviving longer [4]. In that study we found that CIN was associated with increased survival in both landmark and out-of-landmark populations suggesting that CIN absence could be a result of unintended underdosing and that prospective trials were needed to investigate whether drug dosing guided by the occurrence of toxic effects could improve efficacy of standard regimens. The present analysis applies a similar methodology to a larger and more recent population of NSCLC patients receiving chemotherapy within six prospective phase 3 randomized trials. The included clinical trials were similar among themselves, while varied for age, PS and chemotherapy; but these variables were handled through stratification. The landmark population in the present analysis is similar to the landmark population of our 2005 paper, although in the latter median age was higher, male patients and poor PS were slightly more frequent while stage IV was less frequent. Survival was slightly longer in the current population compared with 2005 (median OS 9.8 vs 8.1 months), however patient characteristics and treatment strategies were slightly different justifying some differences in results observed. CIN occurrence was similar to 2005 population, with the worst grade of neutropenia occurring mainly in patients receiving gemcitabine-platinum therapy. The present analysis corroborates the previously observed longer survival in patients with CIN. Importantly, this benefit was more relevant when severe neutropenia was considered. Notably, our findings should not be biased by the use of G-CSF because no prophylactic use was stated by study protocols and these drugs were only adopted in case of grade 4 neutropenia. We also observed that patients developing CIN were younger and, in contrast with 2005 analysis, older age was associated with a greater mortality in the landmark population, although this was not maintained in the out-of-landmark population. However, elderly people may have an increased risk of malnutrition, poor PS, comorbidities and impaired organ function that together with the age per se may negatively affect prognosis. Yet, in contrast with 2005 analysis, female sex here emerged as a significant prognostic factor. In some studies, women seemed to be at greater risk for developing side effects including neutropenia, however, it remains to be clarified whether this is truly linked to a different drugs metabolisms [2]. In lung cancer, in particular, a clear gender-dependent difference in response rate to drug treatment among patients with NSCLC, not affected by age or smoking status, has been reported [20, 21]. Singh et al. [22] reported higher incidence of chemotherapy-related toxicity, increased response rate and longer survival in women with SCLC compared with man, likely due to different pharmacokinetic or pharmacogenomic profiles. However, given the small number of women enrolled in lung cancer trials a definitive conclusion cannot be done.

Neutropenia or leukopenia experienced during chemotherapy have been reported to be associated with improved clinical outcomes in different tumors [2]. A meta-analysis reported approximately a 30% reduction in mortality for patients with advanced cancer or hematological malignancies with higher grade of neutropenia or leukopenia compared with lower grade or lack of cytopenia [3]. In lung cancer, a retrospective analysis of six clinical trials of patients with advanced NSCLC treated with first line docetaxel-gemcitabine chemotherapy, concluded that patients developing neutropenia had significantly higher response rate, time to tumor progression and OS compared with those without neutropenia [23]. Kishida et al. [17] confirmed that CIN was a predictor of better survival in advanced NSCLC, irrespective of pretreatment neutrophil count. In each of these studies, as well as in our previous pooled analysis [4], CIN (irrespective of its severity) was associated with better prognosis, whereas they failed to demonstrate the association between severity of neutropenia and prognosis.

Previous studies suggested that the occurrence of CIN might be a prognostic marker applicable to both resectable and advanced NSCLC [6, 7]. In particular, patients with early-onset of CIN (within 2 cycles of chemotherapy) had significantly better outcomes compared with patients with late-onset or lack of CIN [6, 7].

Numerous variables including genetic factors, tumor microenvironment, metabolism of drugs may impact on chemosensitivity and treatment tolerability [6]. Genetics has significantly improved our knowledge of variability in drug response [24]. In colon cancer, patients with aberration in metabolizing enzymes of fluoropyrimidines and irinotecan are at greater risk of drug-related side effects [25, 26]. Polymorphisms of the UGT1A1 gene have also displayed an association with tumor response [2, 27]. An association of hepatic efflux genes with irinotecan-induced neutropenia has also been described [28]. Similarly, studies on single-nucleotide polymorphisms (SNPs) in drug metabolizing enzymes and transporter seem to predict the risk of neutropenia in patients under treatment with taxanes [29, 30]. In advanced NSCLC, a retrospective analysis failed to demonstrate a significant association between SNPs in DNA repair genes and neutropenia or survival [8]. Given that in many of these studies, no statistical significance was achieved for SNPs alone without the integration of clinical factors, nomograms accounting for clinical and genetic features have been proposed to individuate patients at high risk of CIN [2]. Based on the above-mentioned considerations, in an era aiming at providing precision medicine, a genotype-directed dosing based on pharmacogenomics testing may represent a crucial element. However, a relevant challenge for clinical practice is the unavailability of clinical guidelines and the required expertise to adjust medication dosage based on genetic results [24]. Even if genotype-driven dosing could be feasible, it needs to be assessed in the context of prospective randomized clinical trials in order to provide evidence supporting its application into clinical practice [24].

Our findings suggest that dosing to achieve CIN may be a pragmatic strategy to be considered for improving outcomes in cancer patients. However, prospective trials have been performed to compare fixed-dose versus toxicity-adjusted dose with contrasting results. Our group [31] reported that adapting the dose of chemotherapy based on the toxicities did not improve ORR, PSF and OS over the fixed dose of chemotherapy as first line treatment in patients with extensive SCLC. In breast cancer, based on retrospective data supporting the concept of dose tailoring according to hematologic nadirs and a phase II feasibility trial [32], a confirmatory phase 3 trial compared standard adjuvant chemotherapy versus tailored dose dense therapy [33] and found a significant difference in event free survival favoring the experimental strategy; also, a predefined subgroup analysis showed that dose tailoring is a feasible strategy in obese patients [33, 34]. Hence, further studies incorporating “dose to neutropenia” across different tumor types are needed to establish its applicability into clinical practice.

## Conclusions

The present study confirms our previous finding that CIN could predict survival in advanced NSCLC, thus supporting the hypothesis that CIN could be used as indicator for dose tailoring strategy. Incorporating known pharmacogenetic variations besides neutrophil count monitoring during treatment can be helpful in designing clinical trials based on dose escalation. Genetic, patient and tumor-related factors should be taken into consideration as well as the type of drugs administered in defining initial doses regimen with further escalation and de-escalation strategies guided by the occurrence of hematological and non-hematological side effects during early cycles. Future prospective trials are needed to confirm these data.

## Supplementary Information

**Additional file 1: Table S1.** Main characteristics of the 6 randomized trials included in the pooled analysis. **Table S2.** Distribution of patients according to trial and treatment in the whole population and in landmark and out-of-landmark populations. **Table S3.** Patient characteristics in the whole eligible population (*N* = 1529) according to neutropenia. **Table S4.** Patient characteristics in the landmark population (*N* = 572) according to neutropenia. **Table S5.** Patient characteristics in the out-of-landmark population (*N* = 957) according to neutropenia. **Table S6.** Worst grade of neutropenia by treatment and trial in the landmark group. **Figure S1.** Distribution of worst grade of neutropenia over cycles in the landmark population.

## Data Availability

The datasets used and/or analyzed during the current study available from the corresponding author on reasonable request.

## Abbreviations

BSA: Body surface area
CALC1-E: Cetuximab in advanced lung cancer in elderly
CALC1-PS2: Cetuximab in advanced lung cancer in adult with performance status 2
CIFN: Chemotherapy-induced febrile neutropenia
CIN: Chemotherapy-induced neutropenia
DFS: Disease free survival
GECO: Gemcitabine-coxib in NSCLC
HR: Hazard ratio
MILES2, MILES3 and MILES4: Multicenter Italian lung cancer in the elderly study
NSCLC: Non-small-cell lung cancer
OS: Overall survival
PS: Performance status
TORCH: Tarceva or chemotherapy
