# Supplementary material for: Chemotherapy-induced neutropenia and treatment efficacy in advanced non-small-cell lung cancer: a pooled analysis of 6 randomized trials
Source: BMC Cancer. 2021 May 14;21:549. doi: 10.1186/s12885-021-08323-4 (PMC8120920; doi:10.1186/s12885-021-08323-4)
Supplement: Supplementary file 1 — Additional file 1: Table S1. Main characteristics of the 6 randomized trials included in the pooled analysis. Table S2. Distribution of patients according to trial and treatment in the whole population and in landmark and out-of-landmark populations. Table S3. Patient characteristics in the whole eligible population (N = 1529) according to neutropenia. Table S4. Patient characteristics in the landmark population (N = 572) according to neutropenia. Table S5. Patient characteristics in the out-of-landmark population (N = 957) according to neutropenia. Table S6. Worst grade of neutropenia by treatment and trial in the landmark group. Figure S1. Distribution of worst grade of neutropenia over cycles in the landmark population. [file 12885_2021_8323_MOESM1_ESM.docx]

**Chemotherapy-induced neutropenia and treatment efficacy in advanced non-small-cell lung cancer: a pooled analysis of 6 randomized trials**

Piera Gargiulo et al.

**SUPPLEMETARY APPENDIX**

**Table S1. Main characteristics of the 6 randomized trials included in the pooled analysis.**

**Table S2. Distribution of patients according to trial and treatment in the whole population and in landmark and out-of-landmark populations.**

**Table S3. Patient characteristics in the whole eligible population (N=1529) according to neutropenia.**

**Table S4. Patient characteristics in the landmark population (N=572) according to neutropenia.**

**Table S5. Patient characteristics in the out-of-landmark population (N=957) according to neutropenia.**

**Table S6. Worst grade of neutropenia by treatment and trial in the landmark group**

**Figure S1. Distribution of worst grade of neutropenia over cycles in the landmark population.**

**Table S1. Main characteristics of the 6 randomized trials included in the pooled analysis.**

| **Trial** | **Reference** | **Years of enrolment** | **Patients (N)** | **Age, years** | **ECOG PS** | **Study design, treatment arms** | **Treatment arms** | **Hematologic assessment** | **Primary endpoint** | **Primary endpoint result** |
| --- | --- | --- | --- | --- | --- | --- | --- | --- | --- | --- |
| CALC-1E | Gridelli. Lung Cancer, 2010 | Nov2005 -Jun2011 | N=58 (concomitant arm=29;  sequential arm=29) | ≥ 70 | 0–1, 2 | Multicenter randomized phase 2 trial in advanced (stage III and IV) NSCLC in elderly patients; 2 treatment arms: concomitant vs sequential | Concomitant arm:  Gem 1200 mg/m2 iv on days 1 and 8, every 21 days (maximum 6 cycles unless PD) + cetuximab at loading dose 400 mg/m2 iv on day 1, followed by 250 mg/m2 weekly until PD  Sequential arm:  Gem 1200 mg/m2 iv on days 1 and 8, every 21 days (maximum 6 cycles unless PD) followed by cetuximab at loading dose 400 mg/m2 iv on day 1, followed by 250 mg/m2 weekly until PD | On day 1 and 8 of each cycle | 1-year survival rate | Survival rates (95% CI) at 1-year were 41.4% (23.5–61.1) for concomitant arm and 31.0% (15.3–50.8) for sequential arm |
| CALC-PS2 | Gridelli. Lung Cancer, 2010 | Nov2005 -Jun2011 | N=42  (concomitant arm=22;  sequential arm=20) | 19-69 | 2 | Multicenter randomized phase 2 trial in advanced (stage III and IV) NSCLC in adult PS2 patients; 2 treatment arms: concomitant vs sequential | Concomitant arm:  Gem 1200 mg/m2 iv on days 1 and 8, every 21 days (maximum 6 cycles unless PD) + cetuximab at loading dose 400 mg/m2 iv on day 1, followed by 250 mg/m2 weekly until PD  Sequential arm:  Gem 1200 mg/m2 iv on days 1 and 8, every 21 days (maximum 6 cycles unless PD) followed by cetuximab at loading dose 400 mg/m2 iv on day 1, followed by 250 mg/m2 weekly until PD | On day 1 and 8 of each cycle | 1-year survival rate | Survival rates (95% CI) at 1-year were 27.3% (10.7–50.2) for concomitant arm and 35.0% (15.4–59.2) for sequential arm |
| GECO | Gridelli et al. Lancet Oncology 2007 | Jan2003 –May2005 | N=400  (A=125; B= 75; C= 126; D= 74) | <70 | 0-1 | Multicentre, prospective, open label, randomized, factorial phase 3 trial in advanced (stage III and IV) NSCLC; 4 treatment groups: group A, B, C, D | Group A: Gem 1200 mg/mg2 on 30-min iv infusion on days 1 and 8 every 21 days + Cis 80 mg/m2 day 1 every 21 days for six cycles  Group B: the same treatments as group A + oral rofecoxib 50 mg per day until PD  Group C: pci Gem 1200 mg/mg2 over 120-min iv infusion on days 1 and 8 + Cis 80 mg/m2 day 1 every 21 days for six cycles  Group D: the same drugs as group C + oral rofecoxib 50 mg per day until PD | On day 1 and 8 of each cycle | OS | Median OS survival was 47 weeks [95% CI 40–55] with pci Gem vs 44 weeks [36–52] with standard gemcitabine, HR of death 0·93 [0·74–1·17], p=0·41).  The two rofecoxib groups were closed early (on Oct 1, 2004) due to withdrawal of the drug because of safety issues. |
| TORCH | Gridelli at al. J Clin Oncol, 2012 | Dec2006-Nov2009- | N=760  (standard arm=380  experimental arm=380) | < 70  (no age limits for Canadian center) | 0-1 | International, multicentre, open-label, randomized, phase 3 trial;  2 treatment arms (1:1 randomization): standard vs experimental arm | Standard arm:  Cis 80 mg/m2 iv on day 1 + Gem 1200 mg/m2 iv on days 1 and 8 every 3 weeks (6 cycles maximum)-> followed at PD by erlotinib 150 mg per day until PD  Experimental arm:  Erlotinib 150 mg per day -> followed at PD by Cis 80 mg/m2 iv on day 1 + Gem 1.200 mg/m2 iv on days 1 and 8 every 3 weeks (6 cycles maximum)* | On day 1 and 8 of each cycle | OS | 536 deaths recorded (263 in the standard and 273 in the experimental). Median survival was 11.6 months (95% CI, 10.2-13.3 months) in the standard arm and 8.7 months (95% CI, 7.4 to 10.5 months) in the experimental arm. Adjusted HR of death in the experimental arm was 1.24 (95% CI, 1.04 to 1.47) |
| MILES 2P | Gridelli et al. J Clin Oncol, 2007 | Jun2002 – Nov2004- | N= 159  (phase 1=19, phase 2=121) | ≥ 70 | 0-1 | Two phase 1/2 studies of cispl+ gem and cis+vin | Phase I portion:  Cis at starting dose of 50 mg/m2 (level 0) + gem 1000 mg/m2 on days 1 and 8 or vin 25 mg/m2 iv on days 1 and 8 at fixed dose. Planned dose level of cis were: 50 mg/m2 (level 0), 60 mg/m2 (level +1), 70 mg/m2 (level +2) and 40 mg/m2 (level-1). In the phase 2 part of the two studies, a two stage, flexible design was applied at the feasible dose of cisplatin in combination with gem or vin | On day 1 and 8 of each cycle | Unacceptable toxicity | Cis was feasible at 60 mg/m2 with gem and 40 mg/m2 with vin. With the former combination , 50 of 60 (83.3%) patients were treated without unacceptable toxicity |
| MILES 3 | Gridelli et al. J Clin Oncol, 2018 | Mar2011-Aug2016 | N=299 | ≥ 70 | 0-1 | Multicenter, randomized phase 3 trial; 2 treatment arms (1:1 randomization): standard vs experimental arm | Standard:  Gem 1200 mg/m2 iv on days 1 and 8 every 3 weeks (6 cycles)  Experimental:  Cis 60 mg/m2 iv on day 1 + Gem 1000 mg/m2 iv on days 1 and 8 every 3 weeks (6 cycles) | On day 1 and 8 of each cycle | OS | The trial was closed prematurely because of slow accrual, but a joint analysis of MILES 3 and MILES 4 allowed to test whether addition of cisplatin to single-agent chemotherapy prolongs survival in elderly with advanced NSCLC who do not have an EGFR mutation. At a median 2-years follow-up, 384 deaths were recorded (200 in the monotherapy arm and 184 in the combination arm). HR was 0.86 (95% CI, 0.70 to 1.05; P = .14), and median OS was 7.5 months (95% CI, 6.2 to 9.5 months) in the monotherapy arm and 9.6 months (95% CI, 8.1 to 11.7 months) in the combination arm. |
| MILES 4 | Gridelli et al. J Clin Oncol, 2018 | Mar2011-Aug2016 | N=232 | ≥ 70 | 0-1 | Multicenter, randomized phase 3 trial; factorial design: 4 treatment arms (randomization 1:1:1:1): arm A, B, C, D | Arm A:  Gem 1200 mg/m2 iv on days 1 and 8 every 3 weeks (6 cycles)  Arm B:  Cis 60 mg/m2 iv on day 1 + Gem 1000 mg/m2 iv on days 1 and 8 every 3 weeks (6 cycles)  Arm C:  Pem 500 mg/m2 alone on day 1 every 21 days (6 cycles)  Arm D:  Pem 500 mg/m2 + Cis 60 mg/m2in day 1 every 21 days (6 cycles) | ARM A/B: on day 1 and 8 of each cycle;  ARM C/D: on day 1 of each cycle | OS | The trial was closed prematurely because of slow accrual, but a joint analysis of MILES 3 and MILES 4 allowed to test whether addition of cisplatin to single-agent chemotherapy prolongs survival in elderly with advanced NSCLC who do not have an EGFR mutation. At a median 2-years follow-up, 384 deaths were recorded (200 in the monotherapy arm and 184 in the combination arm). HR was 0.86 (95% CI, 0.70 to 1.05; P = .14), and median OS was 7.5 months (95% CI, 6.2 to 9.5 months) in the monotherapy arm and 9.6 months (95% CI, 8.1 to 11.7 months) in the combination arm. |
| MILES 2G | Gridelli et al. Lung Cancer, 2008 | Oct2002 – Jun2003 | N=51 | ≥ 70 | 0-1 | Single-stage phase 2 trial | Pci Gem 1200 mg/m2 on days 1 and 8 every 21 days (maximum 6 cycles) | On day 1 and 8 of each cycle | ORR | Two complete and 7 partial responses, for an overall response rate of 17.6% (95% exact CI: 8.4-30.9%) |

*patients included in the experimental arm with erlotinib were excluded from the analysis.

**Abbreviations:** CI= confidence interval; Cis= cisplatin; CT= chemotherapy; ECOG= Eastern Cooperative Oncology Group; G=grade; Gem= gemcitabine; HR= hazard ratio; iv= intravenous; ORR= objective response rate; OS= overall survival; pci= prolonged constant infusion; PD= progression disease; Pem=pemetrexed; Vin= vinorelbin.

**Table S2. Distribution of patients according to trial and treatment in the whole population and in landmark and out-of-landmark populations.**

|  | **Whole eligible population**  **(N=1529)** | | **Landmark**  **population**  **(N=572)** | | **Out-of-landmark**  **population**  **(N=957)** | |
| --- | --- | --- | --- | --- | --- | --- |
| **Treatment**. n (%) |  |  |  |  |  |  |
| CALC1 G+Cet | 51 (3.3) | | 17 (3.0) | | 34 (3.6) | |
| CALC1 G →Cet | 49 (3.2) | | 15 (2.6) | | 34 (3.6) | |
| GECO C+G | 125 (8.2) | | 49 (8.6) | | 76 (7.9) | |
| GECO C+G+Rof | 74 (4.8) | | 29 (5.1) | | 45 (4.7) | |
| GECO C+pciG | 125 (8.2) | | 48 (8.4) | | 77 (8.0) | |
| GECO C+pciG+Rof | 74 (4.8) | | 30 (5.2) | | 44 (4.6) | |
| TORCH C+G | 369 (24.1) | | 154 (26.9) | | 215 (22.5) | |
| MILES2 C+G | 60 (3.9) | | 26 (4.5) | | 34 (3.6) | |
| MILES2 C+V | 61 (4.0) | | 23 (4.0) | | 38 (4.0) | |
| MILES2 pciG | 50 (3.3) | | 19 (3.3) | | 31 (3.2) | |
| MILES3 G | 144 (9.4) | | 43 (7.5) | | 101 (10.6) | |
| MILES3 C+G | 133 (8.7) | | 52 (9.1) | | 81 (8.5) | |
| MILES4 G | 53 (3.5) | | 19 (3.3) | | 34 (3.6) | |
| MILES4 C+G | 54 (3.5) | | 16 (2.8) | | 38 (4.0) | |
| MILES4 C+P | 51 (3.3) | | 19 (3.3) | | 32 (3.3) | |
| MILES4 P | 56 (3.7) | | 13 (2.3) | | 43 (4.5) | |

Abbreviations: Cet=cetuximab; C=cisplatin; G=gemcitabine; pciG=prolonged constant infusion gemcitabine; P=pemetrexed; Rof=rofecoxib; V=vinorelbine.**Table S3. Patient characteristics in the whole eligible population (N=1529) according to neutropenia.**

|  | **G0**  **(N=999)** | **G1-G2**  **(N=263)** | **G3-G4**  **(N=267)** | **P** |
| --- | --- | --- | --- | --- |
| **Age**. median (IQR) | 71.1 (61.9-75.3) | 67.1 (60.4-72.8) | 65.8 (58.0-71.8) | <0.001 |
| **Gender**. n (%) |  |  |  |  |
| Male | 771 (77.2%) | 206 (78.3%) | 195 (73.0%) | 0.284 |
| Female | 228 (22.8%) | 57 (21.7%) | 72 (27.0%) |  |
| **Performance status**. n (%) |  |  |  |  |
| 0-1 | 958 (95.9%) | 257 (97.7%) | 265 (99.3%) | 0.014 |
| 2 | 41 (4.1%) | 6 (2.3%) | 2 (0.7%) |  |
| **Stage**. n (%) |  |  |  |  |
| IIIb | 115 (11.5%) | 29 (11.0%) | 35 (13.1%) | 0.718 |
| IV | 884 (88.5%) | 234 (89.0%) | 232 (86.9%) |  |
| **Histology**. n (%) |  |  |  |  |
| Squamous | 435 (43.5%) | 95 (36.1%) | 89 (33.3%) | 0.007 |
| Non Squamous | 410 (41.0%) | 132 (50.2%) | 132 (49.4%) |  |
| Undefined | 154 (15.4%) | 36 (13.7%) | 46 (17.2%) |  |

**Table S4. Patient characteristics in the landmark population (N=572) according to neutropenia.**

|  | **G0**  **(N=294)** | | **G1-G2**  **(N=135)** | **G3-G4**  **(N=143)** | **P** |
| --- | --- | --- | --- | --- | --- |
| **Age**. median (IQR) | 74.8 (61.2-74.8) | | 67.4 (61.4-72.8) | 65.9 (58.8-72.6) | <0.001 |
| **Gender.** n (%) |  |  |  |  |  |
| Male | 221 (51.8) | | 102 (23.9) | 104 (24.4) | 0.827 |
| Female | 73 (50.3) | | 33 (22.8) | 39 (26.9) |  |
| **Performance status**. n (%) |  |  |  |  | 0.004 |
| 0-1 | 280 (50.3) | | 134 (24.1) | 143 (25.7) |  |
| 2 | 14 (93.3) | | 1 (6.7) | 0 (0.0) |  |
| **Stage**. n (%) |  |  |  |  | 0.823 |
| IIIb | 39 (54.2) | | 15 (20.8) | 18 (25.0) |  |
| IV | 255 (51.0) | | 120 (24.0) | 125 (25.0) |  |
| **Histology**. n (%) |  |  |  |  | 0.944 |
| Squamous | 111 (52.1) | | 47 (22.1) | 55 (25.8) |  |
| Non Squamous | 140 (50.9) | | 69 (25.1) | 66 (24.0) |  |
| Undefined | 43 (51.2) | | 19 (22.6) | 22 (26.2) |  |

**Table S5. Patient characteristics in the out-of-landmark population (N=957) according to neutropenia.**

|  | **G0**  **(N=705)** | **G1-G2**  **(N=128)** | **G3-G4**  **(N=124)** | **P** |
| --- | --- | --- | --- | --- |
| **Age**. median (IQR) | 71.2 (62.4- 75.4) | 66.6 (58.9- 72.9) | 65.7 (57.6- 70.2) | <0.001 |
| **Gender**. n (%) |  |  |  |  |
| Male | 550 (78.0%) | 104 (81.3%) | 91 (73.4%) | 0.316 |
| Female | 155 (22.0%) | 24 (18.8%) | 33 (26.6%) |  |
| **Performance status**. n (%) |  |  |  |  |
| 0-1 | 678 (96.2%) | 123 (96.1%) | 122 (98.4%) | 0.457 |
| 2 | 27 (3.8%) | 5 (3.9%) | 2 (1.6%) |  |
| **Stage**. n (%) |  |  |  |  |
| IIIb | 76 (10.8%) | 14 (10.9%) | 17 (13.7%) | 0.631 |
| IV | 629 (89.2%) | 114 (89.1%) | 107 (86.3%) |  |
| **Histology**. n (%) |  |  |  |  |
| Squamous | 324 (46.0%) | 48 (37.5%) | 34 (27.4%) | <0.001 |
| Non Squamous | 270 (38.3%) | 63 (49.2%) | 66 (53.2%) |  |
| Undefined | 111 (15.7%) | 17 (13.3%) | 24 (19.4%) |  |

**Table S6. Worst grade of neutropenia by treatment and trial in the landmark group**

|  | **G0** | **G1** | **G2** | **G3** | **G4** |
| --- | --- | --- | --- | --- | --- |
| **Treatment**. n (%) |  |  |  |  |  |
| CALC1 G+Cet | 15 (88.2) | 1 (5.9) | 1 (5.9) | 0 (0.0) | 0 (0.0) |
| CALC1 G →Cet | 10 (66.7) | 0 (0.0) | 0 (0.0) | 5 (33.3) | 0 (0.0) |
| GECO C+G | 24 (49.0) | 7 (14.3) | 8 (16.3) | 8 (16.3) | 2 (4.1) |
| GECO C+G+Rof | 12 (41.4) | 1 (3.4) | 4 (13.8) | 9 (31.0) | 3 (10.3) |
| GECO C+pciG | 21 (43.8) | 4 (8.3) | 5 (10.4) | 12 (25.0) | 6 (12.5) |
| GECO C+pciG+Rof | 8 (26.7) | 3 (10.0) | 6 (20.0) | 9 (30.0) | 4 (13.3) |
| TORCH C+G | 63 (40.9) | 17 (11.0) | 32 (20.8) | 26 (16.9) | 16 (10.4) |
| MILES2 C+G | 14 (53.8) | 2 (7.7) | 3 (11.5) | 5 (19.2) | 2 (7.7) |
| MILES2 C+V | 10 (43.5) | 3 (13.0) | 3 (13.0) | 5 (21.7) | 2 (8.7) |
| MILES2 pciG | 8 (42.1) | 4 (21.1) | 3 (15.8) | 3 (15.8) | 1 (5.3) |
| MILES3 G | 33 (76.7) | 0 (0.0) | 5 (11.6) | 4 (9.3) | 1 (2.3) |
| MILES3 C+G | 29 (55.8) | 2 (3.8) | 10 (19.2) | 9 (17.3) | 2 (3.8) |
| MILES4 G | 16 (84.2) | 1 (5.3) | 1 (5.3) | 1 (5.3) | 0 (0.0) |
| MILES4 C+G | 10 (62.5) | 0 (0.0) | 2 (12.5) | 3 (18.8) | 1 (6.3) |
| MILES4 C+P | 11 (57.9) | 2 (10.5) | 2 (10.5) | 1 (5.3) | 3 (15.8) |
| MILES4 P | 10 (76.9) | 0 (0.0) | 3 (23.1) | 0 (0.0) | 0 (0.0) |
| **Total** | **294 (51.4)** | **47 (8.2)** | **88 (15.4)** | **100 (17.5)** | **43 (7.5)** |

Abbreviations: Cet=cetuximab; C=cisplatin; G=gemcitabine; pciG=prolonged constant infusion gemcitabine; P=pemetrexed; Rof=rofecoxib; V=vinorelbine.

**Figure S1. Distribution of worst grade of neutropenia over cycles in the landmark population.**
